# Comparison of the clinical outcomes between vascularized bone graft and the Masquelet technique for the reconstruction of Gustilo type III open tibial fractures

**DOI:** 10.1186/s12891-022-06010-4

**Published:** 2022-12-01

**Authors:** Ching-Yu Lan, Po-Hao Lien, Yu-Te Lin, Cheng-Hung Lin, Chung-Cheng Hsu, Chih-Hung Lin, Shih-Heng Chen, Yi-Hsun Yu

**Affiliations:** 1 Department of Plastic and Reconstructive Surgery, Chang Gung Memorial Hospital, Chang Gung University and Medical College, Fu-Hsin St. Kweishan, 33302 Taoyuan, Taiwan; 2 Department of Orthopedic Surgery, Musculoskeletal Research Center, Chang Gung Memorial Hospital, Chang Gung University and Medical College, 5, Fu-Hsin St. Kweishan, 33302 Taoyuan, Taiwan

**Keywords:** Open tibial fracture, Bone defect, Vascularized bone graft, Masquelet technique, Induced membrane technique, Union time, Infection

## Abstract

**Background:**

Gustilo type III tibial fractures commonly involve extensive soft tissue and bony defects, requiring complex reconstructive operations. Although several methods have been proposed, no research has elucidated the efficacies and differences between vascular bone graft (VBG) and the Masquelet technique (MT) to date. We aimed to evaluate and compare the clinical effectiveness of VBG and the MT for the reconstruction of Gustilo type III tibial fractures.

**Methods:**

This retrospective cohort study enrolled patients who underwent reconstruction for Gustilo type III tibial fractures using VBG or the MT in a single center from January 2000 to December 2020. The patients’ demographics, injury characteristics, and surgical interventions were documented for analysis. The clinical outcomes including union status, time to union, postoperative infections, and the causes of union failure were compared between the two groups.

**Results:**

We enrolled 44 patients: 27 patients underwent VBG, and 17 underwent MT. The average union time was 20.5 ± 15.4 and 15.1 ± 9.0 months in the VBG and MT groups, respectively (p = 0.232). The postoperative deep infection rates were 70.4% and 47.1% in the VBG and MT groups (p = 0.122), respectively. Though not statistically significant, the VBG group had a shorter union time than did the MT group when the bone defect length was > 60 mm (21.0 ± 17.0 versus 23.8 ± 9.4 months, p = 0.729), while the MT group had a shorter union time than did the VBG group when the bone defect was length < 60 mm (17.2 ± 5.6 versus 10.7 ± 4.7 months, p = 0.067).

**Conclusions:**

VBG and MT are both promising reconstruction methods for Gustilo type III tibial fractures. VBG appears to have more potential in reconstructing larger bone defects, while MT may play an important role in smaller bone defects, severe surgical site infections, and osteomyelitis. Therefore, flexible treatment strategies are required for good outcomes in Gustilo type III open tibial fractures.

## Background

Gustilo type III open tibial fractures are usually severe and complicated, and often require complex reconstruction for extensive soft tissue and bone defects. Of the methods available for managing bone defects such as the conventional bone graft [1] and distraction osteogenesis [2], the use of vascularized bone grafts (VBGs), such as the free fibula flap, and the application of the Masquelet (induced-membrane) technique (MT) are the advanced options for large segmental defects [2].

VBG is performed in a single-stage fashion based on the microsurgical technique of harvesting a bony segment together with the vessels, skin, subcutaneous tissue, and muscles as a composite for the reconstruction of the bone defect and simultaneous wound coverage depending on the injury. The fibula [3], iliac crest [4], and ribs [5] are commonly selected for microsurgical long bone reconstruction with satisfactory results [6, 7].

The MT is performed in two stages [8]. In the first stage, the bone defect is debrided, and a cement spacer composed of polymethyl methacrylate (PMMA) is impacted into the defect to allow for the formation of a vascularized membrane. The defect and cement are then stabilized with either internal or external fixation, and the exposed bone, spacer, vessels, tendons, nerves, or other vital structures are simultaneously covered using local or free flaps. About 6 to 8 weeks are required for the formation of a mature and vascularized membrane around the cement. In the second stage, the membrane is incised, the cement is carefully removed before filling the defect with an autologous bone graft, typically taken from the iliac crest as in the present study, and definitive internal fixation using intramedullary nails or plates is performed [8].

To date, a consensus on which method is better indicated for open tibial fractures with bone defects is still under debate. In the present study, we aim to analyze the outcomes of VBG and the MT used for the reconstruction of the bone defect of Gustilo III open tibial fractures by reviewing two decades of cases treated in a single tertiary trauma center and to determine the most suitable reconstructive methods.

## Methods

We enrolled 483 consecutive patients with open tibial fractures from a single tertiary trauma center between January 2000 and December 2020. Of the 483 patients, 42, 107, and 334 patients had Gustilo-Anderson types I, II, and III tibial fractures [9], respectively. The inclusion criteria were patients with Gustilo type III tibial fractures who underwent VBG or the MT for the reconstruction of the bone defect caused by trauma, whereas the exclusion criteria included defects related to malignancy or other non-trauma causes and patients that underwent treatment other than the MT or VBG. Finally, 44 patients with type III traumatic open tibial fractures who underwent reconstruction of the bone defects using VBG or the MT were included in the study.

The 44 patients were retrospectively reviewed and divided into two groups: the VBG and MT groups. A total of 27 patients who underwent VBG were compared with 17 patients who underwent MT. All 44 patients received proper initial management for severe trauma or multiple trauma on arrival at the hospital. The bone and soft tissue defects were managed after the general conditions were stabilized.

The operative protocol [6] of the VBG transfer was established, and the free fibular osteoseptocutaneous flaps were the top priority for reconstruction except in patients with bilateral fibular fractures or failure of the fibular flaps. When the wounds were relatively clean or could be obliterated with a single composite bone flap, a single-stage reconstruction was performed. However, in the case of extensive lesions and intractable wound infection, a two-stage reconstruction was performed, namely soft-tissue reconstruction first and a VBG transfer after stabilization of the wound. The operative protocol of the MT [8] was enacted as a two-stage reconstruction. In the first stage, radical debridement of all the traumatized tissues was performed, and a PMMA cement spacer was inserted into the segmental bone defect. An external or internal fixator was used for 6 to 8 weeks generally. During the second stage, the spacer was carefully removed to protect the induced bioactive membrane. Definitive internal fixation, either by plating or nailing, was performed after removing the bone cement. The autologous non-vascularized bone graft (NVBG) from the iliac crests was the main material used to fill the bone defect. Repeated autologous NVBG transplantations were performed to facilitate fracture union in patients with delayed union or non-union after the MT or VBG.

Age, sex, body mass index, fracture type, fracture site, associated injury, size of the bone defect, preoperative and postoperative infection, comorbidity, and type of flap were recorded. The durations of debridement and sequestrectomy, NVBG, from injury to soft tissue coverage and osteosynthesis, and union were evaluated. The Mangled Extremity Severity Score (MESS) and AO soft tissue grading system were used to quantify the severity of soft tissue injuries in the cases with open tibial fractures. The MESS reflects the perfusion of the leg and the mechanism of injuries [10], whereas the AO soft tissue grading system provides more details on the injured soft tissues including the skin, muscles, tendons, nerves, and vessels [11]. The Injury Severity Score (ISS) revealed the severity of multiple traumas.

Radiographic evaluations were performed using the picture archiving and communication system. The final length of the defect was evaluated by reviewing the films after definitive fixation in both groups. We defined the bone defect length as the average length of the largest cortex defects observed in the anterior–posterior and lateral views, since bone defects were well-circumscribed after sequestrectomy and definitive internal fixation in both groups, and the largest cortex defects often serve as a marker of the largest distance that must be bridged during the bone healing process. Fracture union was considered when the radiographic union score for tibial fractures [12], measured by three board-certified orthopedic surgeons, was ≥ 10. The union time was the interval between definitive osteosynthesis to bone union. If the surgeons had similar interpretations, the mean union time was recorded. If there was a difference in the interpretation but two surgeons had similar interpretations, the differences were overlooked, and the result was reported. However, if all the surgeons provided different interpretations, another senior surgeon (Y.-H. Y.) interpreted the images to determine the final result. Despite the consensus that union times exceeding 6–9 months and 12 months are traditionally regarded as delayed union and non-union, respectively, most of the patients in our cohort had a union time of more than 1 year. Therefore, to differentiate cases with a union time of more than 1 year from cases of non-union, non-union was defined as the absence of bony union or limited progress of union observed on X-ray at the time when the patient discontinued care at our hospital after more than 1 year of follow-up. Delayed union was defined as a union time of more than 1 year. Such union time stratification was done to fulfill the outcome analyses for type III tibial fractures in the present study [13].

The diagnosis of post-operative infection was based largely on the criteria defined by the Centers for Disease Control and Prevention [14], clinical symptoms, and laboratory tests. The clinical symptoms of post-operative infection included local erythema and swelling of the surgical wound, pus or pus-like drainage, unexplained fever after the index surgery, poor wound healing, and formation of a draining sinus from the surgical wound(s) that required debridement and intravenous antibiotics. The laboratory signs included leukocytosis (white blood cell count more than 11,000 × 10^9^ cells/L) with a left shift, elevated levels of C-reactive protein more than 5 [15], and positive wound culture results from the surgical wound(s).

### Statistical analysis

The statistical analyses were performed using SAS version 9.1.3 (SAS Institute Inc., Cary, NC, USA). The associations among categorical variables were analyzed using the chi-square test or Fisher’s exact test. The means of continuous variables were compared using the independent t-test or Wilcoxon rank-sum test, and the medians of continuous variables were compared using the Mann–Whitney U test. The Shapiro–Wilk test was used to check whether data were normally distributed. If the p value was less than 0.05, indicating that the variable was not normally distributed, the Wilcoxon rank-sum test was performed; otherwise, the independent t-test was performed.

## Results

**Fig. 1:**
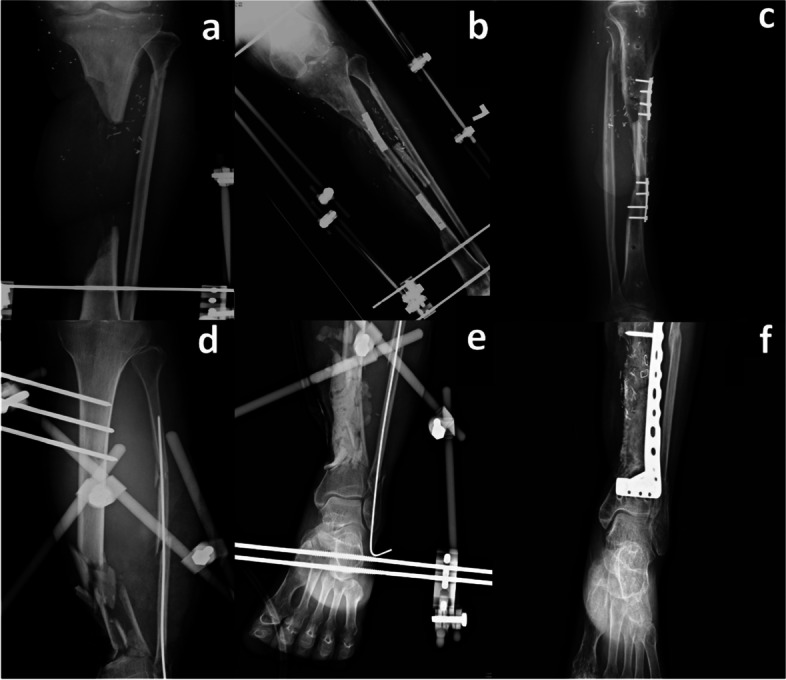
Radiographic images of pre-, post-operative, and united IIIC fractures. **a d** Preoperative tibial fractures with temporary external fixators; **b e** Postoperative images: **b** vascularized bone graft fixed with plate, **e** Masquelet stage I with cement spacer; **c f** United tibial fractures

A total of 44 patients, 34 men and 10 women, were eligible for this study. The age range was 12–69 (37.41 ± 15.43) years. The VBG group had a relatively younger age than did the MT group (33.44 ± 13.65 and 43.71 ± 16.37 years, respectively). Furthermore, 27 patients underwent reconstruction using VBG including 18 free fibular osteoseptocutaneous flaps, 6 free iliac crest flaps, 1 free medial epicondylar bone flap, and 2 free latissimus dorsi flaps with anterior serratus muscle and ribs, whereas 17 patients underwent two-stage MT surgeries. The patient demographics are demonstrated in Table 1. Additionally, of the 27 VBG cases, 20 (74.1%) underwent single-staged VBG reconstruction, while seven (25.9%) underwent two-staged reconstruction. These seven cases included three cases of extensive soft tissue loss and four cases of intractable wound infection that required prior local flaps, free flaps, prolonged application of external skeletal fixation, or intravenous antibiotic treatment. Figure 1 shows pre-operative, post-operative, and united fracture radiographs from our cohort.

**Table 1:** Demographics, fracture patterns, and trauma severity of the cohort

	Overall N = 44 (%)	Masquelet N = 17 (%)	Vascularized bone N = 27 (%)	p-value
Age (mean ± SD)	37.41 ± 15.43	43.71 ± 16.37	33.44 ± 13.65	0.03
Sex				0.473
Female	10 (22.7%)	5 (29.4%)	5 (18.5%)	
Male	34 (77.3%)	12 (70.6%)	22 (81.5%)	
BMI (mean ± SD)	25.32 ± 4.40	25.88 ± 3.86	24.94 ± 4.77	0.487
Fracture type				0.521
IIIA	4 (9.1%)	2 (11.8%)	2 (7.4%)	
IIIB	23 (52.3%)	7 (41.2%)	16 (59.3%)	
IIIC	17 (38.6%)	8 (47.1%)	9 (33.3%)	
Fracture site				0.577
Proximal metaphysis	10 (22.7%)	3 (17.6%)	7 (25.9%)	
Diaphysis	20 (45.5%)	7 (41.2%)	13 (48.1%)	
Distal metaphysis	14 (31.8%)	7 (41.2%)	7 (25.9%)	
Bone gap (mean ± SD)	74.83 ± 42.67	51.16 ± 16.23	94.94 ± 47.97	0.001
With Fibula	42 (95.5%)	17 (100.0%)	25 (92.6%)	0.515
Vascular or nerve injury	18 (40.9%)	9 (52.9%)	9 (33.3%)	0.255
MESS (mean ± SD)	4.50 ± 1.41	4.94 ± 1.20	4.22 ± 1.48	0.099
AOIO*				0.102
3	17 (38.6%)	4 (23.5%)	13 (48.1%)	
4	27 (61.4%)	13 (76.5%)	14 (51.9%)	
AOMT*				0.431
3	15 (34.1%)	7 (41.2%)	8 (29.6%)	
4	29 (65.9%)	10 (58.8%)	19 (70.4%)	
AONV*				0.244
1	23 (52.3%)	7 (41.2%)	16 (59.3%)	
2	2 (4.5%)	0 (0.0%)	2 (7.4%)	
3	19 (43.2%)	10 (58.8%)	9 (33.3%)	
ISS > 16	7 (15.9%)	3 (17.6%)	4 (14.8%)	0.863
DM**	2 (4.5%)	1 (5.9%)	1 (3.7%)	0.736
HTN***	2 (4.5%)	1 (5.9%)	1 (3.7%)	0.736
Smoking	20 (45.5%)	6 (35.3%)	14 (51.9%)	0.283
Infection before surgery	26 (59.1%)	11 (64.7%)	15 (55.6%)	0.548

^*^AOIO: AO soft-tissue grading system of open skin injury; AOMT: AO soft-tissue grading system of muscle/tendon injury; AONV: AO soft-tissue grading system of neurovascular injury; BMI: body mass index; **DM: diabetes mellitus; ***HTN: hypertension; ISS: Injury Severity Score; MESS: Mangled Extremity Severity Score; SD: standard deviation

One patient in the MT group requested and underwent below-knee amputation. This patient underwent the first stage of the MT surgery but refused to undergo the second stage due to the time-consuming and painful nature of the reconstruction process, which was scheduled for 6 months after the first stage.

The present study included Gustilo-Anderson type IIIA, IIIB, and IIIC tibial fractures with bone defects, and the fracture locations were classified into the tibial plateau, proximal metaphysis, diaphysis, and distal metaphysis. There was no significant difference between the MT and VBG groups regarding the location of the bone defect and Gustilo classification.

As shown in Table 1, the length of the tibial bone defects ranged from 26.2 to 204.8 mm, and the mean was 74.83 ± 42.67 mm. The bone defect was significantly larger in the VBG group than in the MT group (94.94 ± 47.97 mm versus 51.16 ± 16.23 mm, p = 0.001). The MESS ranged from 3 to 9 (4.50 ± 1.41) in the whole cohort. Four patients in the VBG group and three in the MT group had multiple traumas with an ISS of more than 16 points. There was no significant difference between the two groups regarding the MESS, AO soft-tissue grading, and ISS.

All the patients in the VBG group used free flaps for bone reconstruction and soft tissue coverage of the exposed bone, implant, tendon, and neurovascular structures. Although no osseous free flap was used in the MT group, soft tissue free flaps were used in 14 patients (82%) for the reconstruction of extensive soft tissue defects and coverage of the bone or implant (Table 2).

**Table 2:** Methods of soft tissue coverage

	Masquelet N = 17	Vascularized bone N = 27	p-value
Flap			0.051
Local flap	3 (17.6%)	0 (0.0%)	
Free flap	14 (82.4%)	27 (100.0%)	

Continuous: mean ± standard deviation with independent t-test

Categorical: by chi-square test or Fisher’s exact test

The development of osteomyelitis was observed in 12 (44.4%) of 27 cases and six (35.3%) of 17 cases in the VBG and MT groups, respectively, and the mean procedural times for debridement or sequestrectomy in the two groups were 3.1 ± 2.6 and 2.2 ± 1.9 (p = 0.455), respectively. In total, 11 (40.7%) and five (29.4%) cases in the VBG and MT groups, respectively, required additional NVBG (p = 0.531), while 12 (44.4%) and 14 (82.4%) cases underwent internal fixator reapplication (p = 0.026). The mean union time for these cases was 24.3 ± 9.2 months in the VBG group and 13.8 ± 12.1 months in the MT group (p = 0.090).

### Union time

The mean union time was 20.5 ± 15.4 months in the VBG group and 15.1 ± 9.0 months in the MT group (Table 3). Seven (25%) patients in the VBG group and eight (47%) in the MT group achieved bone union within 1 year, whereas 14 (52%) and 7 (41%) patients in the VBG and MT groups, respectively, had delayed union. There were no significant differences regarding union within 1 year, delayed union, and nonunion between the two groups.

**Table 3:** Outcomes: union status and postoperative infection

	Masquelet N = 17	Vascularized bone N = 27	p-value
Union status			0.150
Union within 1 year	8 (47.1%)	7 (25.9%)	
Delayed union	7 (41.2%)	14 (51.9%)	
Nonunion	2 (11.7%)	6 (22.2%)	
Average union time (months)	15.1 ± 9.0	20.5 ± 15.4	0.232
Infection after operation	8 (47.1%)	19 (70.4%)	0.122
Post-op debridement & sequestrectomy	2.00 ± 1.70	2.56 ± 2.24	0.193

Continuous: mean ± standard deviation with independent t-test

Categorical: by chi-square test or Fisher’s exact test

In the VBG group, six patients had bone defects greater than 100 mm (average: 154.4 ± 43.4 mm; range: 106.3–204.8 mm) and the union time was relatively longer (average: 22.4 ± 25.0 months; range: 4.7–72.1 months). In addition, five patients with a union time of more than 2 years (average: 22.9 ± 17.3 months; range: 26.6 – 72.1 months) had osteomyelitis.

In the MT group, all cases underwent internal fixation. Nine cases (52.9%) were fixed with plates while eight (47.1%) were fixed with nails, and the union time was 12.3 ± 6.4 and 18.2 ± 10.9 months (p = 0.217), respectively. Additionally, two patients had a union time of more than 2 years (37.1 and 27.8 months, respectively), one of whom was the only patient with diabetes in our cohort; this patient had complications caused by osteomyelitis and underwent repeated debridement and cement spacer placement. The other patient had no perioperative infections or co-morbidities and had relatively large bone defects of 65 mm.

**Fig. 2:**
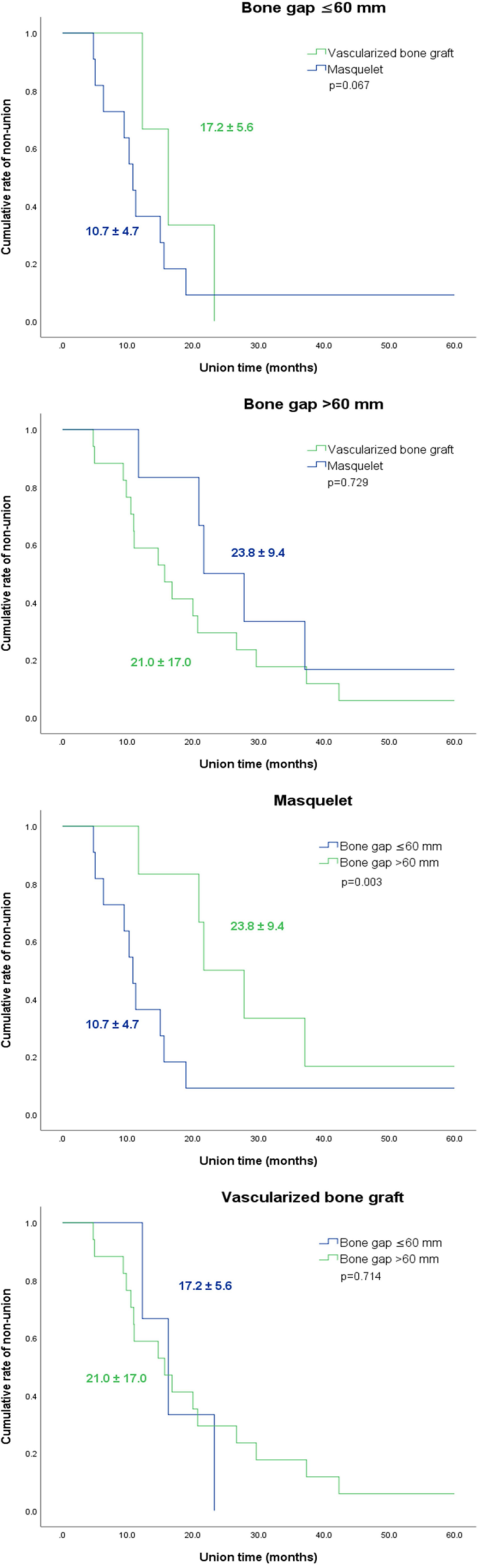
Kaplan–Meier plots for union time in both groups. Subgroup analysis was performed regarding bone defects shorter or longer than 60 mm

We further divided the two groups into subgroups based on bone defect length (every 10 mm) in order to evaluate the difference in the union time between VBG and the MT. Bone defect lengths were classified as greater than or less than 60 mm to elaborate the relationship between defect length and union time, and statistical significance was determined using the Kaplan–Meier method as shown in Fig. 2. The union time for defects less than 60 mm was not significantly different between the VBG and MT groups (17.2 ± 5.6 months versus 10.7 ± 4.7 months, p = 0.067). The union time for defects greater than 60 mm also was also not statistically different between groups, despite bone defects being relatively shorter in the VBG group than in the MT group (21.0 ± 17.0 months versus 23.8 ± 9.4 months, p = 0.729) (Table 4).

**Table 4:** Literature review of the outcome studies regarding MT and VBG

Study, year of publication	Type IIIB and C / all tibial fractures	Bone defect (cm) mean (range)	Acute reconstruction/after nonunion	Union time (months) mean (range)	Major adverse outcome	Post-operative deep infection
**Masquelet technique**						
Kang et al. [20]	15/15	5.8 (4 – 11)	15/0	N/A (4–7)	0 (0%)	0 (0%)
Sasaki et al. [28]	1/5	4.9 (2.5 – 6)	0/5	6 (4–9)	0 (0%)	1 (20%)
Zoller et al. [19]	3/8	6 (3 – 10)	4/4	13.5 (1.8–27)	4 (50%)	5 (62.5%)
Ma et al. [29]	6/15	0.97 (0 – 3.5)	0/15	7.5 (3–12)	0 (0%)	3 (20%)
Cho et al. [30]	8/11	7.52 (3.4–15.9)	0/11	9.1 (6–12)	2 (18.1%)	0 (%)
Giannoudis et al. [31]	2/11	4.49 (3.5 – 7.5)	4/7	5.7 (2–12)	1 (9%)	1 (9%)
Gupta et al. [32]	3/9	5.26 (3.3 – 8.5)	0/9	10.5 (8–13)	1 (11.1%)	2 (22.2%)
**Vascularized bone graft**						
Bibbo et al. [24]	8/8	10.2 (8.9–12.6)	8/0	9 (7–14)	0 (0%)	0 (0%)
Cavadas et al. [22]	41/41	N/A (4 – 17)	9/32	N/A (5–9)	0 (0%)	2 (4.8%)
Zhen et al. [23]	28/28	15 (12 – 18)	28/0	8 (6.5–10.3)	0 (0%)	0 (%)

MT: Masquelet technique, VBG: vascular bone graft

When only the VBG group was analyzed, there was no significant difference in union time for patients with any size of bone defect. In contrast, in the MT group, the union time was significantly shorter in patients with bone defects less than 60 mm than in patients with bone defects greater than 60 mm (10.7 ± 4.7 months versus 23.8 ± 9.4 months, p = 0.003) (Fig. 2).

### Predisposing factors of delayed union and osteomyelitis

Regarding union within 1 year or delayed union, only cigarette smoking was a predisposing factor for delayed bone union after tibial reconstruction using the MT or VBG in the univariate analysis (with an odds ratio of 0.25 for union within 1 year, p = 0.045, using the chi-square test). However, no significant factors affecting union were identified in the multivariate analysis.

More than half of the patients in both groups had preoperative infection, including 15 (56%) patients in the VBG group and 11 (65%) in the MT group. Although not statistically significant, more patients in the VBG group had postoperative infection than in the MT group (19 [70%] versus 8 [47%], p = 0.122). Furthermore, 12 (44.4%) of 27 and 6 (35.3%) of 17 patients in the VBG and MT groups, respectively, had osteomyelitis during the treatment course and follow-up. In the univariate and multivariate analyses, no significant predisposing factor for osteomyelitis was found.

## Discussion

Open type III tibial fractures are often complicated by soft tissue and bone defects and are commonly associated with infection including osteomyelitis. To properly manage these clinical complications, various techniques including the VBG and MT have been proposed (Table 4). However, the two techniques have not been compared to date. In the present study, there was no statistical difference between the VBG and MT groups regarding union time, total treatment time, and subsequent osteomyelitis. In the VBG group, the union time was relatively constant regardless of the defect length, whereas in the MT group, the union rate was significantly better in the patients with bone defects less than 60 mm than in the patients with bone defects greater than 60 mm. Thus, we propose a 60 mm cut-off point for the two reconstruction methods. VBG may serve as the more reliable technique in cases where the bone defect length is greater than 60 mm, while MT may be applied in cases where bone defects are less than 60 mm or in cases with severe surgical site infection or osteomyelitis. We speculate that the reason VBG presented with better outcomes for larger defects might be due to the blood supply being completely and immediately transplanted to the fracture site, while the blood supply of MT relies on the angiogenesis process and therefore the bone healing process may potentially be limited by the bone defect length.

For the past two decades, there were two major surgical strategies for treating large tibial bone defects in our hospital: the MT and VBG. The MT has a low incidence of donor bone morbidity [16], low infection rate [16], high union rate [16], and is suited for various lengths of defects [2]. However, a long and multi-step treatment course was the main drawback of this technique [2]. In addition, the microsurgical technique for soft tissue reconstruction was inevitable in approximately 40% of patients in a meta-analysis [16] and 82.4% in the current study. On the contrary, simultaneous reconstruction of both the bone and soft tissue defects using VBG, a single-stage surgery, may shorten the treatment course. Although various VBG sources have been proposed, the free fibular flap is still the main workhorse considering the length and strength of the graft [6], and subsequent NVBGs are required in some cases including infection or delayed union [3, 6, 7].

Although the mean defect size was greater in the VBG group, the union time was comparable in both groups, especially in patients with bone defects less than 60 mm. Regarding the patients with bone defects greater than 60 mm, the bone defects seemed to heal faster in the VBG group than in the MT group, although this difference was not statistically significant. This was probably related to some extreme cases (bone defect greater than 100 mm) in the VBG group leading to a prolongation in the union time. No study has investigated and compared VBG and the MT when bone defects are present. Only a few studies have compared the two treatment options with other reconstructive methods independently. In a study by Klifto et al. [17], traumatic tibial diaphyseal defects were grouped into < 5 cm, 5–10 cm, and > 10 cm, and VBG resulted in favorable outcomes in the 5–10 cm and > 10 cm groups compared with the NVBG and bone transport techniques. On the contrary, the MT had better functional outcomes but a similar union rate when compared with the bone transport technique, and the average bone defect length was 7 cm [18]. However, the bone defect length in most of the studies that reported an average union time within 1 year using the MT was only approximately 5 cm.

Although bone reconstruction using the MT has been developed for more than two decades, it was only applied in our hospital over the last 5 years. Our results revealed that the mean union time was 10.7 ± 4.7 months for bone defects less than 60 mm and 23.8 ± 9.4 months for bone defects greater than 60 mm. In the MT group, the average union time was 15.1 ± 9.0 months (4.7–27.8 months), and the mean bone defect length was 51 mm (26–80 mm). Zoller et al. [19] reported eight tibial defects treated with the MT for indications including type IIIB fractures and nonunion with or without infection. The average union time was 13.5 months measured from the second stage of the MT to radiographic union. Kang et al. revealed that the union time ranged from 4 to 7 months after the second stage of the surgery [20]. The union time was shorter than that reported by Zoller and the present study. The difference might be due to postoperative osteomyelitis. Only one postoperative superficial infection without deep infection or osteomyelitis was reported in Kang’s series; however, the rates of postoperative osteomyelitis reported by Zoller and the current study were 62.5% and 47.1%, respectively. Therefore, an aseptic environment during bone grafting in the second stage of the surgery of the MT might decrease the union time and final union. This was similar to the results of Masquelet [21]. In addition, Masquelet et al. [8] also proposed in a review article that the healing of bone defect is independent of the defect size, which echoed and reinforced our finding that bone defect length might not be a predisposing factor of delayed union or non-union.

In contrast, VBG has been an irreplaceable method for large bone defect reconstruction since the discovery of microsurgery in the 1970s [2] and has been applied in our hospital since the early 90 s [6]. Cavadas et al. [22] reported that the time needed to achieve full weight-bearing ranged from 9 to 14 months. Zhen et al. [23] and Bibbo et al. [24] reported that the average union time was 8.5 months and 9.4 months using 27 and 8 fibular flaps, respectively. We suggested that the longer union time in our study compared with that in literature was due to the extreme nature of the cases in our cohort. The union time of five patients in the VBG group was more than 2 years. All five patients had severe osteomyelitis or deep infection that required repetitive sequestrectomies and several revisions of internal fixation after the VBG transfer. In addition, one of the five cases had a 20-cm bone defect, and two had multiple traumas with an ISS of > 16 points, which may have prolonged the union time [25]. In the three aforementioned studies, no patient presented with a 20-cm bone defect, and the presence of multiple traumas were not specified, which might have contributed to the difference in the union time between the present study and the other studies.

Although considerable efforts were made to avoid bias during this comparative study, the study still has some limitations. First, the present study was retrospective and nonrandomized, and the long study period could have led to a bias in the surgical protocols. In addition, the limited number of patients in the present study limited individual matching comparisons between the two groups. However, only very few studies have compared the efficacy of bone reconstruction techniques, such as bone graft versus bone transport [26], vascularized bone flap versus NVBG [27], and the MT and other reconstructive procedures [16]. In the present study, the MT was compared with the VBG for the reconstruction of bone defects in Gustilo type III open tibial fractures. The strength of the current study was that the bone union time, preoperative and postoperative infections, the interval from injury to soft tissue coverage or osteosynthesis, and the time of NVBG were analyzed. Although the univariate and multivariate analyses did not reveal significant prognostic factors for bone union or postoperative infection, the optimal surgical technique depending on the length of the bone defect was revealed.

## Conclusions

In conclusion, both the VBG and MT are good and reliable options for the reconstruction of Gustilo type III open tibial fractures. The union time was comparable between the MT and VBG in patients with bone defects less than 60 mm; moreover, the efficacy of the vascularized bone flap reconstruction does not decrease even with a large bone defect reaching 200 mm. Considering the complicated and unpredictable nature of the injuries during trauma, adaptable and flexible treatment strategies or even the combination of two methods for the reconstruction of Gustilo type III open tibial fractures can probably achieve better outcomes.

## Data Availability

The datasets used and/or analyzed during the current study are available from the corresponding author on reasonable request.

## Abbreviations

ISS: Injury Severity Score
MESS: Mangled Extremity Severity Score
MT: Masquelet technique
NVBG: Non-vascularized bone graft
PMMA: Polymethyl methacrylate
VBG: Vascularized bone graft
